# Lower-Grade Gliomas: An Epidemiological Voxel-Based Analysis of Location and Proximity to Eloquent Regions

**DOI:** 10.3389/fonc.2021.748229

**Published:** 2021-09-21

**Authors:** Tomás Gómez Vecchio, Alice Neimantaite, Alba Corell, Jiri Bartek, Margret Jensdottir, Ingerid Reinertsen, Ole Solheim, Asgeir S. Jakola

**Affiliations:** ^1^Department of Clinical Neuroscience, Institute of Neuroscience and Physiology, University of Gothenburg, Sahlgrenska Academy, Gothenburg, Sweden; ^2^Department of Neurosurgery, Sahlgrenska University Hospital, Gothenburg, Sweden; ^3^Department of Neurosurgery, Karolinska University Hospital, Stockholm, Sweden; ^4^Department of Clinical Neuroscience, Karolinska Institute, Stockholm, Sweden; ^5^Department of Neurosurgery, Rigshospitalet, Copenhagen, Denmark; ^6^Department of Health Research, SINTEF Digital, Trondheim, Norway; ^7^Department of Circulation and Medical Imaging, Faculty of Medicine and Health Sciences, Norwegian University of Science and Technology, NTNU, Trondheim, Norway; ^8^Department of Neuromedicine and Movement Science, Faculty of Medicine and Health Sciences, Norwegian University of Science and Technology, NTNU, Trondheim, Norway; ^9^Department of Neurosurgery, St. Olavs Hospital, Trondheim University Hospital, Trondheim, Norway

**Keywords:** glioma grade 2, glioma grade 3, surgical oncology (Mesh), diagnostic imaging—methods, magnetic resonance imaging—methods, neurologic deficit

## Abstract

**Background:**

Glioma is the most common intra-axial tumor, and its location relative to critical areas of the brain is important for treatment decision-making. Studies often report tumor location based on anatomical taxonomy alone since the estimation of eloquent regions requires considerable knowledge of functional neuroanatomy and is, to some degree, a subjective measure. An unbiased and reproducible method to determine tumor location and eloquence is desirable, both for clinical use and for research purposes.

**Objective:**

To report on a voxel-based method for assessing anatomical distribution and proximity to eloquent regions in diffuse lower-grade gliomas (World Health Organization grades 2 and 3).

**Methods:**

A multi-institutional population-based dataset of adult patients (≥18 years) histologically diagnosed with lower-grade glioma was analyzed. Tumor segmentations were registered to a standardized space where two anatomical atlases were used to perform a voxel-based comparison of the proximity of segmentations to brain regions of traditional clinical interest.

**Results:**

Exploring the differences between patients with oligodendrogliomas, isocitrate dehydrogenase *(IDH)* mutated astrocytomas, and patients with *IDH* wild-type astrocytomas, we found that the latter were older, more often had lower Karnofsky performance status, and that these tumors were more often found in the proximity of eloquent regions. Eloquent regions are found slightly more frequently in the proximity of *IDH*-mutated astrocytomas compared to oligodendrogliomas. The regions included in our voxel-based definition of eloquence showed a high degree of association with performing biopsy compared to resection.

**Conclusion:**

We present a simple, robust, unbiased, and clinically relevant method for assessing tumor location and eloquence in lower-grade gliomas.

## Introduction

Glioma is the most common intra-axial tumor, and its location relative to eloquent areas of the brain is important for treatment decisions (1–3). Diffuse lower-grade gliomas (dLGGs) are preferentially located in functional areas near primary eloquent areas of the human brain (4, 5). Besides playing an important role in clinical management of adult patients with glioma, tumor location is also linked to the underlying tumor biology (6, 7).

Epidemiological studies often report tumor location crudely based on anatomical taxonomy, while eloquence often is classified using the University of California San Francisco (UCSF) classification (8) or the classification by Sawaya (9). Such methods require considerable knowledge of neuroanatomy by the rater and add a degree of subjectivity to the evaluation. Yet, identification of eloquent areas (8, 10) is key for dLGG treatment management, and an unbiased and reproduceable method to determine eloquence is desirable.

A robust classification of eloquence could be of high value to surgeons prior to surgery, facilitate risk assessment, and be useful for assessing the need for functional diagnostic work-ups (e.g., functional magnetic resonance imaging (fMRI), diffusion tensor imaging (DTI), or transcranial magnetic stimulation (TMS)), and for assessing the need for intraoperative mapping or monitoring. A robust estimation of proximity to areas of assumed eloquence that is measurable in all patients would also be of importance for research purposes. More overreaching, the strength of this epidemiological approach is to gain awareness of commonly eloquent areas under dLGG influence. Areas often encountered should be emphasized in teaching and surgical training. Neurorehabilitation may design programs based upon regions most often involved and potentially injured by tumor growth or treatment. Also, neuroscientists may find this epidemiological approach useful to understand which regions that frequently can be studied in patients with slow-growing brain tumors in studies on, for instance, region-specific plasticity and specific functional networks. Recently, a tool using normative data in glioblastomas was published where surgeons can upload patient images and get information of involved areas and expected surgical results based upon historical data (1). The use of larger dLGG datasets with richer clinical variables may open the way to develop a similar tool for patients with dLGG.

In this study, we aim to report a voxel-based method where we assess overall dLGG distribution and proximity to critical eloquent regions of interest to neurosurgeons. This will provide an epidemiological background on dLGG predilection sites and proximity to eloquent areas of well-established relevance. To demonstrate the relevance of the model, we will present preliminary data on the association of location with surgical treatment and tumor biology. The chosen software and parameter settings are fully disclosed.

## Material and Methods

### Study Design

This study was produced in the frame of a collaboration between three neurosurgical departments in Norway and Sweden. Patients screened for inclusion were 18 years or older and underwent primary surgery (either biopsy or resection) in the time period 2010–2018. Included patients had a histopathological verified supratentorial diffuse glioma World Health Organization (WHO) grade 2 or 3. We chose to include WHO grades 2 and 3 tumors as they usually have no significant edema causing additional mass effect and distortion of anatomy.

### Data Collection

Clinical and radiological data were retrieved from the electronic health records (EHR) at each institution or collected from research projects conducted locally. Pseudonymized data from each institution were gathered for analysis. Clinical data included patient demographics, the Karnofsky performance score (KPS) (11), symptoms at presentation, histopathological tumor grade, *isocitrate dehydrogenase (IDH)* and chromosomal arms 1p and 19q status (1p19q codeletion or intact), main tumor location, tumor largest diameter, and presumed eloquence based on UCSF criteria. A cohort consisting of 343 patients was curated for analysis.

Histopathological analyses and molecular evaluations were conducted locally, either as clinical practice or reclassified for research purposes following the 2016 World Health Organization classification of tumors of the central nervous system (WHO 2016) (12). Mutational status of *IDH* was assessed with immunohistochemistry staining and next-generation sequencing; 1p19q codeletion was evaluated with fluorescence *in situ* hybridization or methylation array (13, 14). In a minority of cases, reclassification according to the WHO 2016 was not possible due to lack of tissue.

The main tumor location and the largest diameter were registered from anatomical magnetic resonance imaging (MRI) using T2 weighted image (T2) or fluid-attenuated inversion-recovery (FLAIR) sequences. Location taxonomy followed the anatomical lobe mainly involved by the lesion. Multifocal lesions were classified according to the largest tumor. Presumed eloquent brain areas were identified preoperatively following the areas listed in the USCF LGG score (8).

Radiological data included preoperative MRI acquired at different hospitals (1.5T and 3.0T scanners). Scanners were routinely maintained by the vendors; sequences were originally optimized at the respective hospitals as part of the clinical preoperative work for clinical evaluation of brain lesions. Technical data such as scan vendors, software releases, and image acquisition parameters were not acquired for this study. Sequences gathered for this study included T1 weighted image (T1), T1 with gadolinium postcontrast (T1c), T2, and FLAIR. Since all sequences were not available for all patients, only patients with key pair sequences of either T2 or FLAIR, and either T1 or T1c, were included in the analysis. T2 and FLAIR sequences had a mean voxel size of 0.7 mm (0.4–1.2 min–max) for both axes in the axial plane and a mean slice thickness of 3.5 mm (0.5–7.0 min–max), while 43% of these sequences had a voxel size equal or inferior to 1 mm^3^, 27% had voxel size between 1 and 2 mm^3^, and 30% had a voxel size over 2 mm.

### Semi-automatic Annotations

Digital Imaging and Communications in Medicine (DICOM) data of all sequences were converted to Neuroimaging Informatics Technology Initiative file format (NIfTI) with the software 3D Slicer (15). Several trained raters segmented the tumors based upon T2 or FLAIR images. All segmentations were produced in a semi-automatic manner on a case-by-case basis using the tools “Paint,” “Draw,” and “Level tracing” from the Module “Segment Editor” and exported as binary label maps in the 3D Slicer.

All segmentations were further validated by a neurosurgeon (AJ) with long experience in LGG management and research, including volumetric assessment. Raters performing segmentations were blinded to the clinical status of the subjects at the time of the tumor segmentation. Since diffuse glioma WHO grades 2 or 3 infrequently has significant surrounding edema, hyperintense areas on the T2 or FLAIR sequence were considered as tumor invaded. In exceptional cases, attributable edema areas without convincing signs of tumor invasion were excluded from the segmentation.

### Preprocessing

Standard preprocessing was done with Functional Magnetic Resonance Imaging of the Brain Software Library (FLS) (16) as follows: all sequences for a given patient were registered to the T2 or FLAIR image (matching the modality selected for segmentation).

The registered T1 or T1c images were then individually registered to the Montreal Neurological Institute (MNI) space, for which the T1 symmetric MNI 09a was used as the registration target (17). Tumor segmentations and T2 or FLAIR images were then transformed to the MNI space by applying the transformation matrix generated during T1 or T1c registration to the MNI space. All tumor segmentations and T2 or FLAIR images transformed to the MNI space were individually controlled for errors or unexpected deformations by a single rater with experience in glioma image analysis (TG).

Image registration was performed using 12 parameter affine transformations in FSL’s Functional Magnetic Resonance Imaging of the Brain Linear Image Registration Tool (FLIRT). All registration parameters are provided in the **Supplementary Material**.

### Anatomical Atlases, Eloquence, and Neuropsychological Regions of Interest

In order to assess the tumor proximity to the regions of interest (ROIs), two anatomical atlases were used. A well-known probability atlas of three-dimensional reconstructed white matter tracs (18) was included together with the recently released Cerebrum Atlas (CerebrA)—a cortical and subcortical parcellation atlas (19).

Grounded on traditional clinical interest in the neurosurgical community, we focused on regions based on *a priori* anatomical identification and on relevant regions identified previously in documented intraoperative mappings. Traditionally, eloquent parcellated areas include the basal ganglia, visual cortex, and Broca’s and Wernicke’s areas (8, 20). These anatomical regions were mapped to the following parcellations: precentral, pars opercularis, pars triangularis, postcentral, supramarginal and inferior parietal, and pericalcarine area. Due to the frequent involvement of the medial temporal lobe and the importance of this area in memory and learning, we also included the hippocampus and the parahippocampal area. The corresponding subcortical white matter tract anatomy previously reported in the literature (21–23) were included: corticospinal (CS); perisylvian anterior, posterior and long components of the superior longitudinal fasciculus (SLF) with a separate report for arcuate fasciculus (AF); inferior fronto-occipital fasciculus (IFOF); and the optic radiations (OR). Inferior parietal, supramarginal, pars triangularis, pars opercularis, SLF, AF, and IFOF were only considered eloquent and analyzed when involvement was on the left side due to their involvement in language most often being left lateralized.

### Automated Calculation and Statistical Analysis

For each registered spatial segmentation of the patient’s tumor, we computed the extent of the segmentation overlapping the ROIs (accounting for both white matter tracts and parcellated areas). To assess the presence of a white matter tract in a given voxel of the probability atlas, we chose a likelihood above 50% (24–27). A minimum overlapping volume of 1 mm^3^ between the ROI and tumor segmentations was considered as proximity to the ROI in this study. To check the robustness of results, a sensitivity analysis applying a threshold of 10 mm^3^ was used in one application.

To assess the tumors in the proximity of the ROIs, an automated calculation of individual tumor segmentation, anatomical parcellations, and white matter tract overlap was produced. It included a binary identification of the overlapping ROIs, the volume of the overlapped area, and the volumes of the tumor and ROIs in the MNI space. Calculations were performed in Python using NumPy (28) and SimpleITK (29) libraries. In the **Supplementary Material**, the code structure for calculations is presented.

Heatmap visualizations were generated in the 3D Slicer (15). All tumor location maps are three-dimensional. However, illustrations for the tumor location heatmaps are represented showing axial and coronal slices. For visualization purposes, the heatmaps were normalized by computing the cumulative number of observed segmentations for each voxel and divided by the total amount of cases in that group (N). Circular bar plots were generated in Python using NumPy, Pandas (30), and Matplotlib (31).

Analysis of the result of Python calculations and patient data was conducted in IBM SPSS version 28 (IBM Corp., Armonk, NY, USA). Central tendencies are presented either with percentages, means with 95% confidence interval (CI), or medians with quartiles 1 and 3 (Q1, Q3). All tests were two-sided; the statistical significance level was set to P < 0.002 due to multiple testing. Comparisons between groups were conducted with unpaired t-test, Mann–Whitney U test, and Fisher’s exact test or one-way ANOVA, Kruskal–Wallis test, and Fisher–Freeman–Halton exact test when appropriate. Interrater reliability analysis was performed using Cohen’s Kappa statistic. Univariable logistic regression was used in consecutive test with choice of primary surgical strategy and binarized KPS as response. Age at surgery, preoperative KPS, tumor volume, tumor classification, preoperative eloquence, and voxel-based eloquence were used as dependent variables.

## Results

### Patient Characteristics

**Figure 1:**
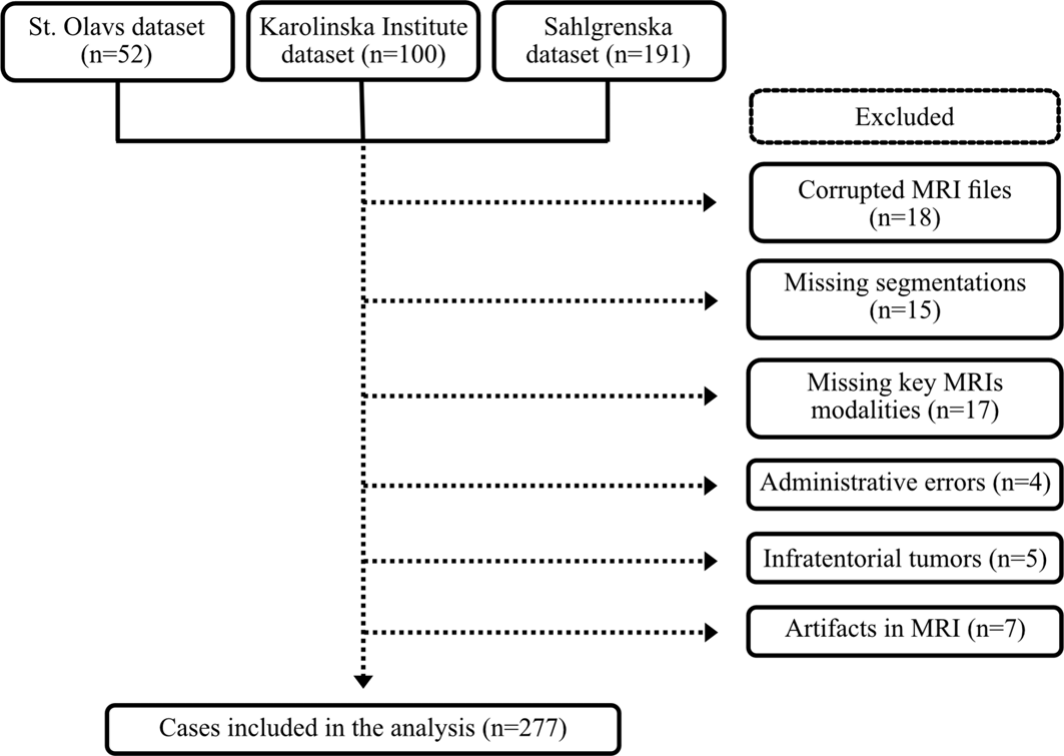
Flow chart of included cases.

A total of 343 patients were eligible for inclusion in this study. Subsequently, 61 cases were excluded due to administrative or technical problems together with 5 cases with infratentorial tumors; see **Figure 1**. Thus, 277 patients were included in the analyses. The mean age at surgery was 45.1 ± 14.7 years, and 160 (57.8%) patients were males.

We had complete molecular data according to WHO 2016 in 222 cases (80.1% of total). There were 73/277 (26.3%) patients with oligodendroglioma, 67/277 (24.2%) were *IDH*-mutated astrocytoma, and 82/277 (29.6%) were *IDH* wild-type astrocytoma. The remaining 55 cases (19.9%) were not characterized molecularly. In total, 182/277 (65.7%) were WHO grade 2, and 95/277 (34.3%) were WHO grade 3. Demographic distribution, histomolecular data, and clinical variables of the study cohort are shown in **Table 1**.

**Table 1:** Clinical characteristics of the studied population.

Variable	Cohort (N = 277)
Age at surgery, mean (95% CI)	45.1 (43.4 - 46.8)
Female, No (%)	117 (42.2)
KPS^1^ at admission, median (Q1, Q3)	90 (80, 90)
Time from radiological diagnosis to surgery in months, median (Q1, Q3)	1 (1-3)
WHO^2^ grade 2, No (%)	182 (65.7)
WHO grade 3, No (%)	95 (34.3)
WHO 2016^3^, No (%)	
Oligodendroglioma	73 (26.3)
*IDH*-mutated astrocytoma	67 (24.2)
*IDH* wild-type astrocytoma	82 (29.6)
Not characterized molecularly	55 (19.9)
Asymptomatic, No (%)	19 (6.9)
Epilepsy, No (%)	181 (65.3)
Any focal neurological deficit at admission, No (%)	79 (28.5)
Choice of neurosurgical intervention, No (%)	
Biopsy only	55 (19.9)
Main tumor location, No (%)	
Frontal	148 (53.4)
Insular	21 (7.6)
Occipital	1 (0.4)
Parietal	30 (10.8)
Temporal	65 (23.5)
Central, deep, basal ganglia, or thalamus	12 (4.3)
Presumed eloquence, No (%)	182 (65.7)
Largest diameter in millimeters, mean (95% CI)	52.0 (49.6 - 54.4)
Tumor volume^4^ in ml, median (Q1, Q3)	47.4 (21.5 - 86.4)
Tumor volume^5^ in ml, median (Q1, Q3)	56.9 (27.1 - 105.6)

^1^ Karnofsky performance status. ^2^ World Health Organization. ^3^ 2016 WHO Classification of the Tumors of the Central Nervous System. ^4^ Tumor volumes computed in patient space. ^5^ Tumor volumes computed after registration to MNI space. For convenience, all volumes are reported in milliliters.

**Figure 2:**
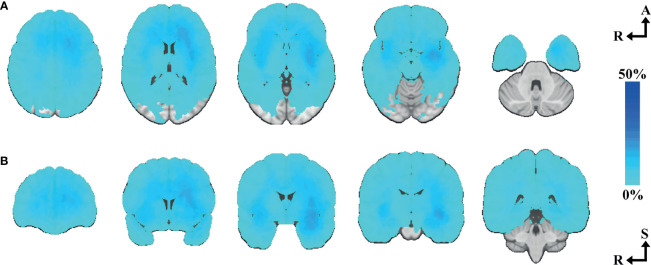
Tumor heatmap of all included cases. **(A)** N=277, axial slices number 33, 15, 0, -15, -33. **(B)** N=277, coronal slices number 40, 15, 0, -15, -40. Axial-coronal coordinates in MNI space. The color intensity represents the voxel-based percentual distribution of the selected tumor segmentations.

**Figure 3:**
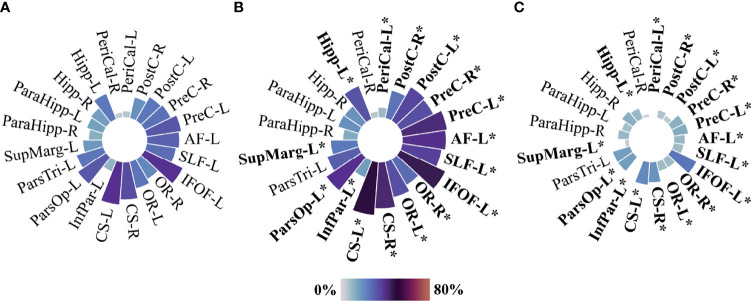
Circular bar plots—proximity to predefined eloquent regions. **(A)** All included cases N=277, **(B)** presumed eloquent N=182, and **(C)** non-eloquent N=95. PreC, Precentral; PostC, Postcentral; PeriCal, Pericalcarine; Hipp, Hippocampus; ParaHipp, Para hippocampus; SupMarg, Supramarginal; ParsTri, Pars Triangularis; ParsOp, Pars Opercularis; InfPar, Inferior parietal; CS, Corticospinal; OR, Optic radiations; IFOF, Inferior fronto-occipital fasciculus; SLF, Perisylvian anterior, posterior, and long components of the superior longitudinal fasciculus; AF, Arcuate fasciculus. “L” and “R” indicate left and right sides, respectively. Size and color intensity represent the percentage of tumors in the proximity to predefined eloquent regions by group. Fisher exact test was used for two-group comparison between Presumed eloquent and Non-eloquent groups. *P value equal or inferior to 0.002.

A location heatmap showing the spatial distribution for all 277 tumors is shown in **Figure 2**. Circular bar plots showing the frequencies of tumors in their proximity to the predefined critical regions are shown in **Figure 3A**. For a description of associations between voxel-based and clinician reported eloquence, see **Supplementary Table 1**.

### Regression Analysis

**Figure 4:**
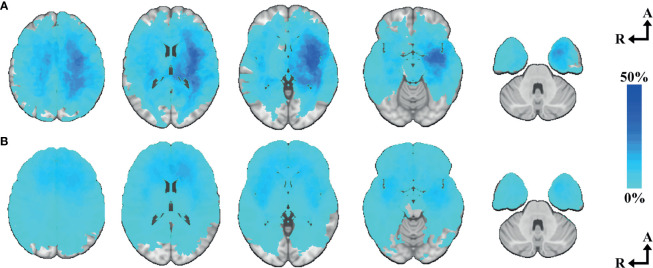
Tumor heatmap by choice of primary surgical strategy. **(A)** Biopsy heatmap N=55—axial slices number 33, 15, 0, -15, -33. **(B)** Resection heatmap N=222—axial slices number 33, 15, 0, -15, -33. Axial coordinates in MNI space. The color intensity represents the voxel-based percentual distribution of each group.

**Figure 5:**
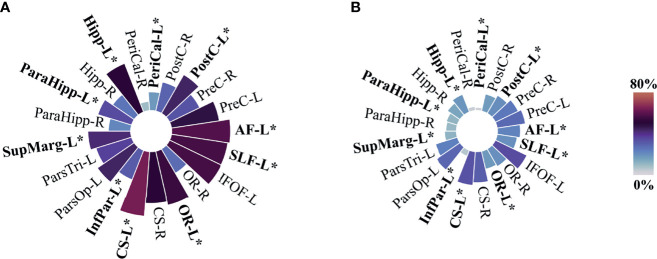
Circular bar plots by choice of primary surgical strategy. **(A)** Only biopsy N=55. **(B)** Tumor resection N=222. PreC, Precentral; PostC, Postcentral; PeriCal, Pericalcarine; Hipp, Hippocampus; ParaHipp, Para hippocampus; SupMarg, Supramarginal; ParsTri, Pars Triangularis; ParsOp, Pars Opercularis; InfPar, Inferior parietal; CS, Corticospinal; OR, Optic radiations; IFOF, Inferior fronto-occipital fasciculus; SLF, Perisylvian anterior, posterior, and long components of the superior longitudinal fasciculus; AF, Arcuate fasciculus. “L” and “R” indicate left and right sides. Size and color intensity represent the percentage of tumors in the proximity to predefined eloquent regions by group. Fisher’s exact test was used for two-group comparison between only biopsied and resected tumors. *P value equal or inferior to 0.002.

Choice of primary surgical strategy in all cases (N=277) was used as the target to evaluate the relevance of the areas included in our voxel-based eloquence. Age, eloquence according to UCSF criteria, and tumor volume were found independent predictors of choice of biopsy as primary surgical strategy (95% CI [0.028 to 0.072], p <0.001; 95% CI [1.313 to 3.701], p <0.001; and 95% CI [2.485E-6 to 9.626E-6], p <0.001, respectively). When applying the regression analysis only in cases characterized molecularly according to WHO 2016 (N=222), *IDH* wild-type astrocytoma was found to be an independent predictor of choice of biopsy as primary surgical strategy (95% CI [1.175 to 3.061], p <0.001). Out of the 21 predefined areas, 10 were significantly associated with performing biopsy instead of resection. Location heatmaps showing the spatial distribution by choice of primary surgical strategy are shown in **Figure 4**. Circular bar plots showing the frequencies of tumors in their proximity to the predefined critical regions are shown in **Figure 5**. A description of frequencies on tumor proximity is shown in **Table 2**.

**Table 2:** Tumor proximity to selected ROI—Comparison by choice of surgical strategy//Sensitivity analysis *with overlap threshold 10 mm3*.

	Population-based (N=277)	Biopsy (N=55)	Resection (N=222)	P value^1^	*Univariable logistic regression*		*Sensitivity analysis*		
					Response: Only biopsy		//Biopsy (N=55)	//Resection (N=222)	//P value^1^
					95% Wald CI	P value^2^			
Any voxel-based eloquent region, No (%)	232 (83.8)	–	–	–	0.400, 3.301	0.012	–	–	–
Cortical and subcortical parcellation atlas, No (%)									
Precentral cortex left	88 (31.8)	26 (47.3)	62 (27.9)	0.009	0.234, 1.444	0.007	26 (47.3)	59 (26.6)	0.005
Precentral cortex right	73 (26.4)	18 (32.7)	55 (24.8)	0.236	-0.250, 1.030	0.232	18 (32.7)	52 (23.4)	0.168
Postcentral cortex left	69 (24.9)	24 (43.6)	45 (20.3)	**<0.001**	0.488, 1.739	**<0.001**	24 (43.6)	43 (19.4)	**<0.001**
Postcentral cortex right	54 (19.5)	16 (29.1)	38 (17.1)	0.057	0.008, 1.365	0.047	16 (29.1)	35 (15.8)	0.032
Pericalcarine left	17 (6.1)	10 (18.2)	7 (3.2)	**<0.001**	0.903, 2.939	**<0.001**	10 (18.2)	6 (2.7)	**<0.001**
Pericalcarine right	14 (5.1)	5 (9.1)	9 (4.1)	0.163	-0.274, 1.997	0.137	4 (7.3)	5 (2.3)	0.080
Hippocampus left	69 (24.9)	28 (50.9)	41 (18.5)	**<0.001**	0.893, 2.149	**<0.001**	27 (49.1)	38 (17.1)	**<0.001**
Hippocampus right	47 (17.0)	14 (25.5)	33 (14.9)	0.072	-0.040, 1.381	0.064	13 (23.6)	33 (14.9)	0.155
Para hippocampal area left	44 (15.9)	19 (34.5)	25 (11.3)	**<0.001**	0.731, 2.120	**<0.001**	17 (30.9)	22 (9.9)	**<0.001**
Para hippocampal area right	35 (12.6)	12 (21.8)	23 (10.4)	0.038	0.110, 1.653	0.025	12 (21.8)	20 (9.0)	0.016
Supramarginal left	49 (17.7)	23 (41.8)	26 (11.7)	**<0.001**	1.016, 2.364	**<0.001**	23 (41.8)	24 (10.8)	**<0.001**
Pars Triangularis left	71 (25.6)	20 (36.4)	51 (23.0)	0.057	0.018, 1.282	0.044	18 (32.7)	48 (21.6)	0.110
Pars Opercularis left	87 (31.4)	24 (43.6)	63 (28.4)	0.035	0.062, 1.277	0.031	24 (43.6)	59 (26.6)	0.021
Inferior parietal left	31 (11.2)	17 (30.9)	14 (6.3)	**<0.001**	1.107, 2.681	**<0.001**	16 (29.1)	12 (5.4)	**<0.001**
Atlas of reconstructed white mater tracs, No (%)									
CS left	108 (39.0)	35 (63.6)	73 (32.9)	**<0.001**	0.656, 1.890	**<0.001**	34 (61.8)	68 (30.6)	**<0.001**
CS right	95 (34.3)	28 (50.9)	67 (30.2)	0.007	0.274, 1.476	0.004	26 (47.3)	63 (28.4)	0.010
OR left	70 (25.3)	30 (54.5)	40 (18.0)	**<0.001**	1.066, 2.329	**<0.001**	27 (49.1)	32 (14.4)	**<0.001**
OR right	59 (21.3)	15 (27.3)	44 (19.8)	0.269	-0.262, 1.096	0.229	12 (21.8)	39 (17.6)	0.444
IFOF left	107 (38.6)	31 (56.4)	76 (34.2)	0.003	0.308, 1.510	0.003	31 (56.4)	74 (33.3)	0.003
SLF left	75 (27.1)	31 (56.4)	44 (19.8)	**<0.001**	1.027, 2.280	**<0.001**	31 (56.4)	42 (18.9)	**<0.001**
AF left	82 (29.6)	32 (58.2)	50 (22.5)	**<0.001**	0.944, 2.187	**<0.001**	32 (58.2)	46 (20.7)	**<0.001**

^1^ Fisher’s exact test was used for comparison between biopsy and resection groups. ^2^ Univariable logistic regression was used with choice of primary surgical strategy as response variable. Bold values indicate significant P value < 0.002.

Binarized preoperative KPS in all cases (N=277) was used as the target to explore associations of the areas included in our voxel-based eloquence and functional status. The KPS cutoff was set to 90 identifying patients with normal performance status or with minor symptoms. In 12 of 21 areas, an association with the KPS score was observed as can be seen in **Supplementary Table 1**.

To rule out that the results were affected by inaccuracies in the registration method, a sensitivity analysis was conducted adjusting the parameters of the computational analysis. Results for this, showing the overlap of tumor location and ROIs, are displayed together with the other results in **Table 2**. In this sensitivity analysis, results were consistent using an overlap threshold of 10 mm^3^; hence, for the rest of the analyses, we used an overlap threshold of 1 mm^3^.

### Presumed Clinical Eloquence Compared to Voxel-Based Eloquence in dLGG

To evaluate the concordance of the voxel-based method with traditional measure of eloquence using the UCSF criteria, an interrater reliability analysis was performed. Voxel-based eloquence was considered positive when any of the predefined eloquent regions were overlapping with the tumor segmentation. When comparing the whole cohort (N=277), the analysis showed fair agreement between preoperative UCSF eloquence and voxel-based eloquence. Interrater reliability analysis is displayed in **Table 3**. Since not all regions of our definition of voxel-based based criteria are included in the UCSF criteria (and *vice versa*), we explored all our predefined regions according to UCSF definition of eloquence in **Figures 3B, C**. A description of frequencies according to UCSF criteria can be found in **Supplementary Table 1**. The pars triangularis, parahippocampal areas, the calcarine area on the right side, and the hippocampus on the right side were not associated with the clinician-reported UCSF criteria of eloquence.

**Table 3:** Preoperative UCSF eloquence compared to voxel-based eloquence in dLGG (N=277).

	Presumed Eloquent (N=182)	Non-eloquent (N=95)	Measure of agreement	P value^1^
Any voxel-based proximity of eloquent regions, No (%)	173 (95.1)	59 (62.1)	0.377	**<0.001**
No voxel-based proximity of eloquent regions, No (%)	9 (4.9)	36 (37.9)		

^1^ Cohen’s Kappa test was used for interrater comparison between preoperative UCSF eloquence and voxel-based eloquence. Bold values indicate significant P value < 0.002.

### Proximity to Eloquent Regions in Molecular Subgroups

**Figure 6:**
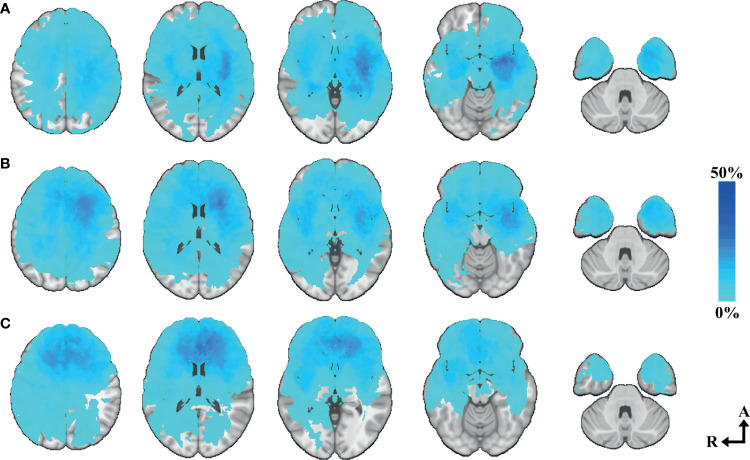
Tumor heatmaps of molecular subgroups according to WHO 2016. **(A)** *IDH* wild-type astrocytomas heatmap N=82—axial slices number 33, 15, 0, -15, -33. **(B)** *IDH*-mutated astrocytomas heatmap N=67—axial slices number 33, 15, 0, -15, -33. **(C)** Oligodendrogliomas N=73—axial slices number 33, 15, 0, -15, -33. Axial coordinates in MNI space. The color intensity represents the voxel-based percentual distribution of each group.

**Figure 7:**
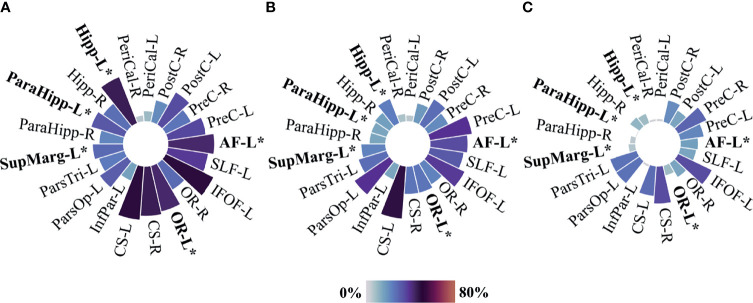
Circular bar plots—proximity to predefined eloquent regions by molecular subgroups according to WHO 2016. **(A)** *IDH* wild-type astrocytomas N=82, **(B)** *IDH*-mutated astrocytomas N=67, and **(C)** Oligodendrogliomas N=73. PreC, Precentral; PostC, Postcentral; PeriCal, Pericalcarine; Hipp, Hippocampus; ParaHipp, Para hippocampus; SupMarg, Supramarginal; ParsTri, Pars Triangularis; ParsOp, Pars Opercularis; InfPar, Inferior parietal; CS, Corticospinal; OR, Optic radiations; IFOF, Inferior fronto-occipital fasciculus; SLF, Perisylvian anterior, posterior, and long components of the superior longitudinal fasciculus; AF, Arcuate fasciculus. “L” and “R” indicate left and right sides. Size and color intensity represent the percentage of tumors in the proximity to predefined eloquent regions by tumor groups. Fisher–Freeman–Halton exact tests were used when appropriate for three-group comparison between *IDH* wild-type astrocytoma, *IDH*-mutated astrocytoma, and oligodendroglioma. *P value equal or inferior to 0.002.

Only tumors classified molecularly according to WHO 2016 were included in the analysis of proximity to eloquent regions in molecular subgroups (N=222). Heatmaps showing anatomical tumor location in patients with *IDH* wild-type astrocytoma either grade 2 or grade 3 (N=82), *IDH*-mutated astrocytoma either grade 2 or grade 3 (N=67), and oligodendroglioma either grade 2 or grade 3 (N=73) are presented in **Figure 6**. Frequencies of proximity to each of the predefined eloquent areas are represented in circular bar plots in **Figure 7**. Details on tumor location and comparative results according to molecular subgroups can be found in **Table 4**. Overall, *IDH* wild-type astrocytomas were more often found in the proximity of the hippocampus, parahippocampal area, optic radiations, and arcuate fasciculus. Involvement of critical eloquent regions was found in a biological gradient.

**Table 4:** Details on voxel-based eloquence and comparative results by molecular subgroups according to WHO 2016.

	Cases with complete molecular data (N=222)	*IDH*-wt astrocytoma (N=82)	*IDH*-mut astrocytoma (N=67)	Oligodendroglioma (N=73)	P value^1^
Clinical variables,					
Age at surgery, mean (95% CI)	46.4 (44.4, 48.3)	52.0 (48.7, 55.3)	40.3 (37.1, 43.4)	45.6 (42.5, 48.7)	**<0.001**
KPS^2^ at admission, median (Q1, Q3)	90 (80, 90)	90 (70, 90)	90 (80, 90)	90 (80, 100)	0.008
KPS (<90), No (%)	83 (37.4)	40 (48.8)	22 (32.8)	21 (28.8)	0.026
Tumor volume^3^ in ml, median (Q1, Q3)	56.9 (27.5, 108.8)	55.1 (19.9, 97.2)	77.9 (32.9, 137.0)	54.2 (28.8, 110.0)	0.185
Presumed eloquence, No (%)	151 (68.0)	62 (75.6)	46 (68.7)	43 (58.9)	0.084
Any voxel-based eloquent region, No (%)	185 (83.3)	77 (93.9)	54 (80.6)	54 (74.0)	0.002
Only biopsy, No (%)	49 (22.1)	35 (42.7)	8 (11.9)	6 (8.2)	<0.001
Cortical and subcortical parcellation atlas, No (%)					
Precentral cortex left	71 (32.0)	29 (35.4)	26 (38.8)	16 (21.9)	0.071
Precentral cortex right	56 (25.2)	21 (25.6)	14 (20.9)	21 (28.8)	0.577
Postcentral cortex left	57 (25.7)	28 (34.1)	18 (26.9)	11 (15.1)	0.022
Postcentral cortex right	44 (19.8)	17 (20.7)	12 (17.9)	15 (20.5)	0.911
Pericalcarine left	14 (6.3)	8 (9.8)	4 (6.0)	2 (2.7)	0.205
Pericalcarine right	12 (5.4)	5 (6.1)	5 (7.5)	2 (2.7)	0.427
Hippocampus left	60 (27.0)	37 (45.1)	16 (23.9)	7 (9.6)	<0.001
Hippocampus right	41 (18.5)	22 (26.8)	11 (16.4)	8 (11.0)	0.036
Parahippocampal area left	37 (16.7)	26 (31.7)	10 (14.9)	1 (1.4)	<0.001
Parahippocampal area right	32 (14.4)	18 (22.0)	10 (14.9)	4 (5.5)	0.010
Supramarginal left	43 (19.4)	23 (28.0)	15 (22.4)	5 (6.8)	0.002
Pars Triangularis left	57 (25.7)	20 (24.4)	18 (26.9)	19 (26.0)	0.944
Pars Opercularis left	70 (31.5)	25 (30.5)	26 (38.8)	19 (26.0)	0.261
Inferior parietal left	25 (11.3)	13 (15.9)	10 (14.9)	2 (2.7)	0.010
Atlas of reconstructed white mater tracs, No (%)					
CS left	92 (41.2)	40 (48.8)	32 (47.8)	20 (27.4)	0.012
CS right	79 (35.6)	37 (45.1)	17 (25.4)	25 (34.2)	0.042
OR left	59 (26.6)	35 (42.7)	17 (25.4)	7 (9.6)	<0.001
OR right	49 (22.1)	23 (28.0)	15 (22.4)	11 (15.1)	0.148
IFOF left	90 (40.5)	40 (48.8)	25 (37.3)	25 (34.2)	0.152
SLF left	63 (28.4)	31 (37.8)	21 (31.3)	11 (15.1)	0.005
AF left	70 (31.5)	35 (42.7)	23 (34.3)	12 (16.4)	0.001

^1^One-way ANOVA, Kruskal–Wallis, or Fisher–Freeman–Halton exact tests were used when appropriate for three-group comparison between oligodendroglioma, IDH-mutated astrocytoma (IDH-mut astrocytoma), and IDH wild-type astrocytoma (IDH-wt astrocytoma). ^2^ Karnofsky performance status. ^3^ Tumor volumes computed after registration to MNI space. Reported in milliliters for convenience. Bold values indicate significant P value < 0.002.

## Discussion

We present a voxel-based method depicting the overall anatomical distribution of dLGG tumors in a population-based sample and their proximity to eloquent regions. As expected, tumor distribution is linked to molecular status. Our voxel-based definition of eloquence had only a fair agreement with clinical reported presumed eloquence using the UCSF criteria. Almost half of the predefined eloquent regions were associated with undergoing biopsy instead of resection, suggesting that our captured regions hold clinical relevance.

Our atlas-based approach to both location and eloquence is based on normative data and not patient-specific functional data. An epidemiological and unselected approach of dLGG relations to subcortical and cortical anatomy mapped to functional data would require DTI and fMRI or TMS to be performed in an unselected manner, which is not a clinical routine in most European specialized centers (32). Such examinations are most often performed when deemed clinically useful on an individual level. Although there exist several methods in use to localize certain functions, there is, however, no perfect match between preoperative functional mapping (fMRI, DTI, TMS), intraoperative mapping/stimulation, and ultimately the final operative result (33–37). There are also discrepancies between dissection-based anatomical studies and DTI studies (38). Our voxel-based mapping of the spatial proximity of the tumor to eloquent cortical regions and related connectivity may provide realistic estimates in an unselected population, although we acknowledge that there are weaknesses at an individual level. Voxel-based mapping, as presented in this study, does not represent an advantage compared to abovementioned methods; however, it allows us to access a larger cohort in an unbiased manner.

With the aim to report a voxel-based method highlighting the overall dLGG anatomical distribution and their proximity to critical regions of interest to neurosurgeons, we showed that eloquent regions are found in a biological gradient that is independent of tumor volume, with most eloquent regions involved in *IDH* wild-type astrocytomas and least eloquent regions involved in oligodendrogliomas. Earlier publications prior to the inclusion of molecular markers in WHO 2016 classification have demonstrated that the frontal, temporal, and insular lobes near eloquent regions are the preferential location of dLGG (39–42). After the inclusion of molecular differentiation for dLGG classification, studies describing the anatomical predilection of *IDH-*mutated subgroups have drawn more detailed conclusions when addressing main lobe location or location in relation to eloquent areas. For instance, studies comparing three molecular subgroups of diffuse gliomas WHO grade 2 (43) reported location in relation to areas of gliogenesis, showing that frontal location was common for *IDH*-mutated tumors without particular differential predilection sites depending on 1p19q status. Others found that 1p19q defined oligodendrogliomas WHO grade 2 were mainly located in the frontal lobes (44), specifically within the deep white matter (45). Studies including patients across all diffuse glioma grades also demonstrated that *IDH*-mutated gliomas are primarily situated in the frontal lobes (46, 47). In clinical practice, *IDH*-mutated tumors had been demonstrated to be more amenable to resection, therefore consistent with the preference of *IDH* wild-type tumors in more critical locations (48, 49). As expected, these studies on dLGG general locations within the brain, together with studies describing more critical location in *IDH* wild-type tumors (50, 51), are in line with our findings. These observations, for instance the more aggressive clinical course in patients with *IDH* wild-type dLGG, make it necessary to reevaluate the extensive literature on residual tumor volume (or extent of resection) and outcome as the historical results may suffer from confounding by tumor biology (12, 52–56).

dLGGs are frequently located in eloquent areas. However, controversies regarding the definition of eloquence have not been settled (57). While the USCF criteria were developed and validated to predict overall survival and progression-free survival (58), application of Sawaya’s grading was found ambiguous due to its definition of the near-eloquent brain (59). Still, the UCSF score and Sawaya’s grading are used as good general predictors of the clinical outcome in patients with dLGG. Our method complements such approaches by including a more detailed account of the involved eloquent regions. Of note, our method is not only reproducible and unbiased but also flexible if the research question makes it relevant to study another area or more specific area of interest (e.g., motor system with supplementary motor area, motor strip and corticospinal tract; language areas for language studies; or hippocampus for memory studies). The strength of our methodological approach is that it is adaptive, where involvement of certain regions can be looked for specifically or in an unselected manner depending on the output of interest. This can be easily done *by replacing our definition of eloquence* with an *a priori* selection of the tracts and cortical areas of interest to the research topic. With more and richer data available, unsupervised analyses may also be of interest to study associations between symptoms or findings with specific areas in more explorative studies.

Preoperative methods to assess eloquence in patients with dLGG are limited and bond to a traditional view of eloquence (60). Compared to other methods, our approach relies on a detailed description of the involvement of the different chosen structures that encode function within the brain. In this study, we proposed a reproducible and objective method, yet dynamic with respect to the chosen area of interest. Further work on this method in relation to tumor remnants and/or neurological and neurocognitive postoperative problems will be explored in future work.

## Strengths and Limitations

A major strength of the present technique is that it is available for all routinely MRI scanned patients. Its simplicity makes it even accessible in context where DTI is not available or when exposure to DTI scanning times is not achievable. The main limitations of the present technique concern the approximation inherent to the atlas approach together with other limitations intrinsic to DTI-tractography techniques (33).

In this report, we aimed to present a general comparison between groups of patients with dLGG. Ideally, and especially in light of 2021 fifth edition of the WHO Classification of the Tumors of the Central Nervous System, a further subtyping of the *IDH* wild-type tumors into glioblastoma or pediatric-type gliomas would be preferable (56). Similarly, the astrocytoma *IDH*-mutant grade 4, as identified through CDKN2A/B homozygous deletions, could preferably have been excluded from our cohort. But since the vast majority of non-enhancing gliomas *IDH* wild type in adults are molecular glioblastomas, and that CDKN2A/B homozygous deletion is rare in the WHO grade 2 group (61), we believe that the results at the group level would not differ from what we have presented.

We chose to include tumor maps of molecular markers as corroboration of findings in previous studies to strengthen the external validity of our model. Despite its simplicity, the linear registration method has been previously benchmarked showing comparably accurate results as nonlinear registration for glioma localization (21, 62). For other aims, a different selection of ROI and/or a more restrictive overlapping threshold could also be applied. In this sense, a voxel-based approach is dynamic but without compromising reproducibility and objectiveness.

A consistent difference can be observed when comparing tumor location heatmaps with tables and plots over the proximity of a tumor to the eloquent areas. While percentages on heatmaps represent voxel-wise overlap, percentages on tables and plots are based on a binary identification of this overlap. Thus, precise quantification of the severity of the presumable lesion caused by the tumor is a remaining challenge. In this study, as a surrogate measure of the lesion, we used a binary identification for when a given ROI was presumably intersected by a tumor. However, this only provides an approximation of the overall involvement of the ROI, and in many instances, this will only mean proximity as tracts may be dislocated rather than infiltrated.

## Conclusion

We have reported overall diffuse lower-grade gliomas (WHO grades 2 and 3) distributions with special emphasis on eloquent areas with a simple and robust method, which may facilitate the reporting of neurosurgical eloquence in an unbiased and comparable, yet dynamic manner.

A biological gradient was observed with most eloquent regions involved in *IDH* wild-type astrocytomas and least critical regions involved in oligodendrogliomas. The regions included in our voxel-based definition of eloquence showed a high degree of association with performing biopsy compared to resection, demonstrating its relevance in clinical practice.

## Data Availability Statement

The raw data supporting the conclusions of this article will be made available by the authors upon reasonable request, without undue reservation.

## Ethics Statement

The studies involving human participants were reviewed and approved by the Regional Committee for Medical and Health Research Ethics in Central Norway (REC reference 2017/1780) and by the Regional Committee of Western Sweden (EPN reference 705/17). All data were pseudo-anonymized; biological material was not shared across institutions. The need for informed consent was waived by both committees. Written informed consent for participation was not required for this study in accordance with the national legislation and the institutional requirements.

## Author Contributions

All authors participated equally in the initial design and final reviewing of the manuscript. TG contributed with planning of the study, data curation, data analysis, and drafting of the manuscript, including design of figures and tables, revising, and submission of the manuscript. AN contributed with data curation, computational scripting and design of figures, reviewing the manuscript, and approval of the final manuscript. AC contributed by recruiting patients, tumor segmentations, reviewing manuscript, and approval of the final manuscript. JB and MJ contributed by recruiting patients, tumor segmentations, reviewing the manuscript, and approval of the final manuscript. OS contributed by planning of the study, recruiting patients, reviewing the manuscript, and approval of the final manuscript. IR contributed with methodological input, reviewing the manuscript, and approval of the final manuscript. AJ contributed with planning of the study, establishing partnerships across institutions, recruiting patients, evaluating the initial tumor segmentations, reviewing the manuscript, and approval of the final manuscript. All authors contributed to the article and approved the submitted version.

## Funding

AJ holds research grants from the Swedish Research Council (2017–00944) and the agreement between the Swedish government and the county councils (the ALF agreement, ALFGBG-716671).

## Conflict of Interest

The authors declare that the research was conducted in the absence of any commercial or financial relationships that could be construed as a potential conflict of interest.

## Publisher’s Note

All claims expressed in this article are solely those of the authors and do not necessarily represent those of their affiliated organizations, or those of the publisher, the editors and the reviewers. Any product that may be evaluated in this article, or claim that may be made by its manufacturer, is not guaranteed or endorsed by the publisher.
