# Supplementary material for: Lower-Grade Gliomas: An Epidemiological Voxel-Based Analysis of Location and Proximity to Eloquent Regions
Source: Front Oncol. 2021 Sep 21;11:748229. doi: 10.3389/fonc.2021.748229 (PMC8490663; doi:10.3389/fonc.2021.748229)
Supplement: Supplementary file 1 [file Table_1.docx]

Supplementary Material

# Supplementary information – Parameter settings for co-registration and registration

*["flirt", "-in", reoriented, "-ref", ref_path, "-omat", transformation_file_path, "-dof", "12", "-cost", "mutualinfo", "-interp", "trilinear"]*

Co-registration 🡪 Input: T1 or T1c and Flair/T2 for each patient case. Method: linear registration of T1 images to Flair/T2 space. Output: T1 images in the same space and resolution as the segmentations. This co-registration target was planned in order to minimize the margin of error considering that the segmentations were based on T2 or FLAIR. Thus, choosing T2 or FLAIR and not T1 or T1c as target, avoided to submit the segmentations through one extra transformation step before the actual transformation to a MNI space (ICBM 2009a Nonlinear Symmetric).

Registration 🡪 Input: Co-registered T1, Flair/T2, segmentation and MNI space template Method: FSL Linear registration of T1s to MNI space, application of transformation matrix on segm and Flair/T2. Output: Segmenations in MNI space (and associated T1, Flair/T2 images in MNI for quality check).

Resampling 🡪 ROIs were resampled with Module “Segment Editor” in 3d Slicer using ICBM 2009a Nonlinear Symmetric as source geometry.

# Supplementary information – Code structure of overlap analysis

Overlap 🡪 Input: ROIs registered tumor segmentation. Method: Overlay each tumor segmentation with each ROI using Python 3. Output: Probability and extent of overlap of each tumor segmentation to each ROI.

*[for every reg_segm:*

*segm_voxel_count = count_nonzero_voxels(reg_segm)*

*for every ROI:*

*overlap = reg_segm*ROI*

*if any non-zero voxel in overlap:*

*overlap_binary = 1*

*overlapping_voxel_count = count_nonzero_voxels(overlap)*

*ROI_voxel_count = count_nonzero_voxels(ROI)]*

# Supplementary Table

**Supplementary Table 1:** Voxel-based eloquence of predefined regions and association with clinician reported eloquence based upon the UCSF reporting system (left side). To the right associations between predefined regions of voxel-defined eloquence with preoperative functional score with a binary dependent variable of KPS <90

|  | ***Presumed Eloquent***  **(N=182)** | ***Non-eloquent***  **(N=95)** | **P value**^1^ | **Univariable logistic regression** | |
| --- | --- | --- | --- | --- | --- |
|  |  |  |  | **Response: KPS <90** | |
|  |  |  |  | **95% Wald CI** | **P value**^2^ |
| Any voxel-based eloquent region, No (%) | 173 (95.1) | 59 (62.1) | **<0.001** | 0.136, 1.756 | 0.022 |
| Cortical and subcortical parcellation atlas, No (%) |  |  |  |  |  |
| Precentral cortex left | 75 (41.2) | 13 (13.7) | **<0.001** | 0.501, 1.563 | **<0.001** |
| Precentral cortex right | 61 (33.5) | 12 (12.6) | **<0.001** | -0.657, 0.490 | 0.776 |
| Postcentral cortex left | 64 (35.2) | 5 (5.3) | **<0.001** | 0.709, 1.844 | **<0.001** |
| Postcentral cortex right | 47 (25.8) | 7 (7.4) | **<0.001** | -0.832, 0.462 | 0.575 |
| Pericalcarine left | 17 (9.3) | 0 (0.0) | **<0.001** | 0.628, 2.781 | 0.002 |
| Pericalcarine right | 11 (6.0) | 3 (3.2) | 0.393 | -0.323, 1.836 | 0.169 |
| Hippocampus left | 59 (32.4) | 10 (10.5) | **<0.001** | 0.873, 2.019 | **<0.001** |
| Hippocampus right | 35 (19.2) | 12 (12.6) | 0.181 | -0.361, 0.939 | 0.384 |
| Parahippocampal area left | 36 (19.8) | 8 (8.4) | 0.015 | 0.762, 2.105 | **<0.001** |
| Parahippocampal area right | 25 (13.7) | 10 (10.5) | 0.568 | -0.674, 0.822 | 0.847 |
| Supramarginal left | 45 (24.7) | 4 (4.2) | **<0.001** | 0.513, 1.778 | **<0.001** |
| Pars Triangularis left | 55 (30.2) | 16 (16.8) | 0.020 | 0.480, 1.596 | **<0.001** |
| Pars Opercularis left | 71 (39.0) | 16 (16.8) | **<0.001** | 0.602, 1.670 | **<0.001** |
| Inferior parietal left | 31 (17.0) | 0 (0.0) | **<0.001** | -0.014, 1.495 | 0.054 |
| Atlas of reconstructed white mater tracs, No (%) |  |  |  |  |  |
| CS left | 88 (48.4) | 20 (21.1) | **<0.001** | 0.817, 1.871 | **<0.001** |
| CS right | 77 (42.3) | 18 (18.9) | **<0.001** | -0.107, 0.934 | 0.120 |
| OR left | 61 (33.5) | 9 (9.5) | **<0.001** | 0.755, 1.888 | **<0.001** |
| OR right | 50 (27.5) | 9 (9.5) | **<0.001** | -0.448, 0.760 | 0.614 |
| IFOF left | 83 (45.6) | 24 (25.3) | **<0.001** | 0.703, 1.750 | **<0.001** |
| SLF left | 66 (36.3) | 9 (9.5) | **<0.001** | 0.665, 1.773 | **<0.001** |
| AF left | 72 (39.6) | 10 (10.5) | **<0.001** | 0.750, 1.838 | **<0.001** |

^1^Fisher’s exact test was used for two groups comparison between presumed eloquent and non-eloquent tumors. ^2^ Univariate logistic regression was used with a binarization of functional score as response variable. Bold values indicate significant P value < 0.002.
